# Application and Outcomes of Indocyanine Green in Endoscopic Transsphenoidal Surgery for Functional Pituitary Tumors: A Literature Review

**DOI:** 10.3390/brainsci16030293

**Published:** 2026-03-06

**Authors:** Moustafa Habes, Damanpreet Kaur Lang, Sami Khairy, Alejandro Vargas-Moreno, Jessica Rabski, Shaun-Jason Kilty, Fahad AlKherayf

**Affiliations:** 1 Faculty of Medicine, University of Ottawa, Roger Guindon Hall, 451 Smyth Rd, Ottawa, ON K1H 8M5, Canada; mhabe005@uottawa.ca; 2 The Ottawa Hospital Research Institute, The Ottawa Hospital, Civic Campus, 1053 Carling Ave., Ottawa, ON K1Y 4E9, Canada; dalang@ohri.ca; 3 Department of Surgery, Division of Neurosurgery, The Ottawa Hospital, Ottawa, ON K1Y 4E9, Canada; skhairy@toh.ca (S.K.); jrabski@toh.ca (J.R.); 4 Department of Otolaryngology—Head and Neck Surgery, University of Ottawa, Ottawa, ON K1H 8L6, Canada; skilty@toh.ca

**Keywords:** functional pituitary tumors, indocyanine green, endoscopic transsphenoidal surgery, intraoperative fluorescence, surgical intervention

## Abstract

**Introduction:** Functional pituitary tumors (FPT) are hormone-producing adenomas that present distinct surgical difficulties, primarily because they are often microadenomas and require total removal to restore normal hormone levels. Distinguishing these tumors from healthy pituitary tissue during endoscopic transsphenoidal surgery can be challenging. Indocyanine green (ICG) fluorescence has shown promise as a technique to enhance visualization during surgery; however, its use in FPTs remains poorly defined. This literature review aims to evaluate the use of ICG in endoscopic transsphenoidal surgery for adult functional pituitary tumors. The primary focus is on assessing complete tumor resection, biochemical remission, and tumor differentiation from normal tissue after ICG use. Secondary goals include reviewing different ICG administration methods. **Methods:** We developed a search strategy to perform a systematic literature search across multiple databases with a set of MeSH terms and keywords on the OVID platform. In addition, we conducted a manual literature search by reviewing the reference lists of the studies included. After this, the relevant search results were uploaded to COVIDENCE for systematic management of the screening process by two reviewers. Articles that met our inclusion criteria were then selected for data extraction. **Results:** The initial search results gave 319 studies, of which 10 met the inclusion criteria after going through various screening phases. These were then included in the final analysis, consisting of 55 adult FPT cases. The majority of studies reported successful differentiation between tumor and normal pituitary tissue, with ICG fluorescence facilitating complete tumor resection and subsequent biochemical remission. Our review also identified two primary ICG administration techniques: low-dose intraoperative injection and high-dose preoperative administration. **Conclusion:** Findings from our study indicate that ICG fluorescence is a clinically effective tool for enhancing tumor visualization during endoscopic transsphenoidal surgery in adult FPT patients. Both low-dose intraoperative and high-dose preoperative ICG administration techniques showed promising outcomes in improving surgical precision. Having said that, further research, with larger comparative studies and the development of a standardized protocol, is required to optimize ICG use in FPTs as well as to address the variability in fluorescence intensity observed among different FPT subtypes.

## 1. Introduction

Pituitary adenomas, the most common subtype of pituitary tumors, are benign neoplasms that originate from adenohypophyseal cells [1]. They are mainly divided into non-functional (non-FPT) and functional tumors (FPT), each with its own unique clinical presentation, local and systemic impacts, complex medical management, and risks of recurrence following treatment [2,3,4]. Non-FPTs are non-hormone-secreting tumors that often cause symptoms when they are large enough to exert pressure on adjacent structures, such as the normal pituitary tissue or the optic chiasm, leading to symptoms like headaches, visual disturbances, or hormonal disturbance due to pituitary compression [5]. On the other hand, FPTs are hormone-secreting tumors that can lead to a multitude of hormonal imbalances depending on the tumor type [2]. The most common type of FPTs are prolactinomas, tumors characterized by excess prolactin secretion, which can present with clinical findings of amenorrhea, galactorrhea, infertility in women, and hypogonadism in men [2,3]. Somatotroph adenomas secrete growth hormone (GH), which leads to acromegaly in adults or gigantism in children. They are subdivided into densely granulated and sparsely granulated subtypes. Corticotroph adenomas secrete adrenocorticotropic hormone (ACTH), resulting in Cushing’s disease. Lastly, Thyrotroph adenomas, although rare, produce thyroid-stimulating hormone (TSH) and lead to hyperthyroidism [2,3].

The treatment strategy for pituitary adenomas relies on observation, medical management, and/or surgical management, depending on the clinical presentation, tumor size, and the type of tumor (functional vs. non-functional) [4]. Non-FPTs are primarily treated by surgical resection, especially when patients present with large masses that are impacting adjacent structures [4,5]. In contrast, treatment of FPT is more variable and often includes more medically targeted therapies, even for patients undergoing surgical resection (pre-op and/or post-op treatment) [4]. Prolactinomas, the most common type of FPT, are commonly treated medically via dopamine inhibitors, as most patients tolerate treatment well and do not require surgical interventions [4,6]. Nonetheless, surgical resection remains the main treatment for patients with other FPTs and patients with prolactinomas that do not tolerate therapy well or are resistant to medical therapy, progressive visual deterioration, and tumor hemorrhage [4,6].

The main treatment for ACTH, GH, and TSH-secreting tumors is surgical resection with the aim of total resection for achieving either long-term cure or control of the disease. Improving intraoperative visualization is a cornerstone in surgeries for FPTs, as they are more likely to be microadenomas (size < 10 mm), which prevent clear presentation in pre-operative imaging, in contrast to non-FPTs, which are more likely to be macroadenomas (size > 10 mm) [7]. Additionally, any residual functional tumor tissue can still cause hormone disruptions post-operatively, thus requiring a careful approach to achieve total tumor resection to avoid post-operative complications and adjuvant treatment [8].

Surgical interventions of pituitary adenomas consist of endoscopic or microscopic transsphenoidal resection of the tumor. These surgical procedures are highly effective and minimally invasive; however, they still have some downsides [9]. Mainly, differentiation between tumor and normal pituitary gland tissue is difficult and is often dependent on the subjective analysis of the surgeon. This can lead to residual tumors with ongoing diseases, which can lead to further morbidity and cost to manage the condition. As a result of this challenge, several methods have been identified to improve the surgical approach to pituitary adenomas, mainly using fluorescent dyes to differentiate between tumor and normal pituitary tissue [7].

One of the most used fluorescent dyes in multiple surgical fields is Indocyanine green (ICG), a water-soluble, tricarbocyanine dye that rapidly binds to plasma proteins upon intravenous injection. When illuminated with near-infrared (NIR) light, ICG emits fluorescence, allowing for real-time visualization of blood flow and tissue perfusion. ICG is a relatively new addition to transsphenoidal pituitary adenoma surgery; it is administered to allow differentiation between tumor and normal pituitary tissue [10,11].

In this review, we will investigate the published literature for exploring the applicability of ICG in endoscopic transsphenoidal surgery for FPT cases, with a primary focus on the utilization and outcome of ICG in achieving tumor-to-gland differentiation and complete resection. As well as the secondary focus will be on evaluating different techniques of ICG administration methods, dosing protocol and timing.

## 2. Methods

We constructed the literature search strategy based on our inclusion and exclusion criteria, without putting restrictions on publication date. Our inclusion criteria were developed in accordance with our primary objective, comprising FPT cases that are classified as follows: growth hormone-secreting adenoma; thyroid-secreting adenoma; microadenomas; macroadenomas; Cushing’s disease; acromegaly; or prolactinoma, in patients undergoing endoscopic transsphenoidal surgery with administration of ICG. The secondary objective, which was also formulated as an additional part of our inclusion criteria, was to identify data on the dose and technique utilized during the ICG administration. The exclusion criteria for our study were: reports using another fluorescence agent in FPT surgeries of adult patients; studies reporting intraoperative utilization of ICG in non-FPT cases or other intracranial malignancies; ICG being used to visualize the surrounding vasculature solely without tumor tissue; or utilization of ICG in endoscopic FPT surgeries of the adult population having secondary malignancies. An additional exclusion criterion was that studies were not included if they did not have full text or results; studies published in a language apart from English, and studies related to animal research.

**Figure 1:**
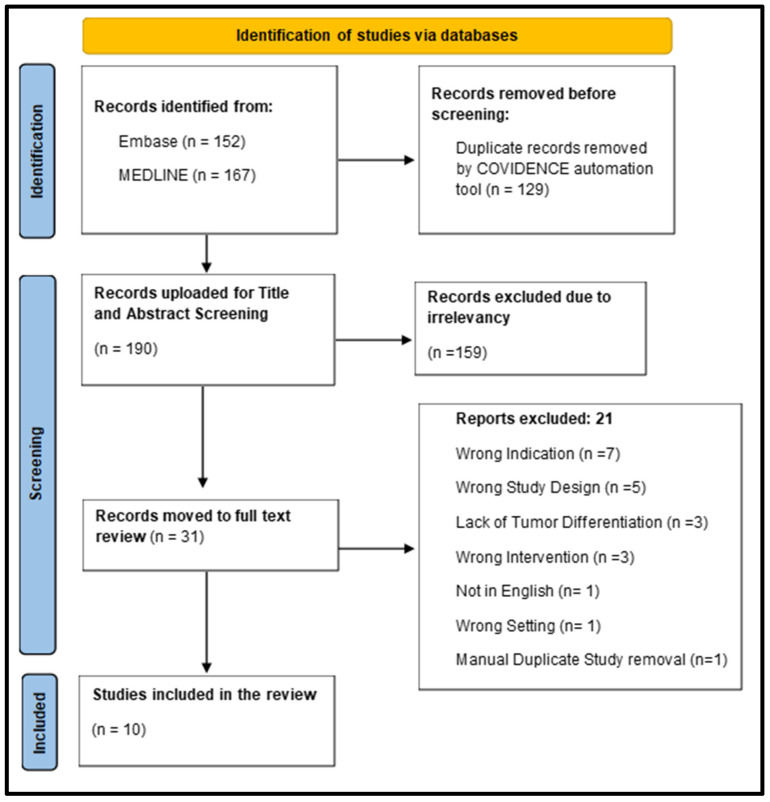
319 records were uploaded to COVIDENCE, out of which 129 duplicates were removed automatically by COVIDENCE. 190 articles screened for Title and Abstract screening with 159 excluded that were not included in our inclusion criteria. 31 full-text articles were screened, and 21 reports were excluded. Data was extracted from 10 articles.

Two electronic databases, Medline and Embase, were used for literature search and to identify relevant studies reported in peer-reviewed journals. Accordingly, the search strategy was formulated for these databases on the OVID platform. The included set of MeSH terms/keywords across the databases was: pituitary neoplasms, pituitary adenoma, functional pituitary adenoma, hormone-secreting pituitary adenoma, prolactinoma, corticotroph, somatotroph, adrenocorticotropic secreting, indocyanine green, endoscopic transsphenoidal surgery, near-infrared fluorescence. In addition to the search strategy mentioned, a manual search from the reference list of included studies was also conducted. The search results were then uploaded to the COVIDENCE platform to systematically manage the screening process by two reviewers consecutively. The platform automatically removed duplicates during title and abstract screening. Each removal was checked and confirmed by both reviewers. Manual removal of duplicates during the screening was not needed. Based on the inclusion and exclusion criteria described above, the selected articles were moved to full-text review and then to the extraction stage. A PRISMA flow diagram is provided to illustrate the screening process (Figure 1).

During this process, 319 studies, including published papers and conference abstracts reporting their results, were uploaded for screening, out of which 129 records were duplicates and were automatically removed by the Covidence platform. A total of 190 studies were moved to the very first stage of screening, which involved the review of titles and abstracts. 159 studies were voted irrelevant by both reviewers, and 31 articles were transferred to the second stage for full-text review. Extending on that, 21 studies were excluded, leaving 10 studies for the data extraction stage (detailed description given in Table 1).

## 3. Results

A total of 10 studies with an overall sample size for the FPT cases from which we drew our results were 55 [12,13,14,15,16,17,18,19,20,21], descriptive analysis given in Table 2. During the full text screening, studies were excluded for the following reasons: 7 published papers had wrong indication such as reporting cases with co-occurrence of cerebral aneurysms, 5 had wrong study design consisting of literature reviews detailing on the biochemistry pathways and pharmacological aspects of ICG; and in vitro studies, in 3 studies the tumor type was not specified, 1 of the study was not published in English (layout given in Figure 1). One excluded study was done using a microscopic transsphenoidal approach, which is a non-relevant setting according to our inclusion criteria. Although we relied on automatic removal of duplicate studies but one of the publications was found to have duplicate results due to being presented as a poster, and we manually excluded it during the full text screening phase. The data that we extracted from the selected papers were the type of pathology, the number of cases that each study investigated and drew their conclusion from, the amount of ICG dose administered, and whether the paper recommends the utilization of ICG in FPT surgeries (Table 1).

**Table 1:** Description of the studies included in the systematic review.

Study	Year of Publication	Sample Size for FPT Cases	Pathology	ICG Administration Summary	ICG Recommended
Wang et al. [21]	2025	1	Cushing’s syndrome	Intraoperative administration of 12.5 mg	Yes
Verstegen et al. [20]	2016	6	Cushing’s Disease; Acromegaly; Prolactinoma	Multiple IV dose of 5 mg ICG with a maximum dose of 15 mg per patient. The first dose was administered before tumor removal, and the and dose at the end of the surgery to check resection.	Yes
Amano et al. [13]	2019	9	Growth Hormone Secreting Adenoma	ICG dose of 6.25 or 12.5 mg was given as a bolus into the peripheral veins.	Yes
Muto et al. [18]	2023	2	Lactotroph Pituitary Adenoma	12.5 mg of ICG was administered intravenously during the surgery.	Yes
Lee et al. [16]	2022	1	GH-secreting Pituitary Adenoma	12.5 mg of ICG was administered intravenously during the surgery.	Yes
Inoue et al. [14]	2015	5	GH and PRL-secreting pituitary adenoma	25 mg of ICG compound was dissolved in 10 mL of sterile water. 5 mL of this solution was administered to patients into the peripheral vein as a bolus during the surgery, followed by 10 mL of saline flush.	Yes
Lindner Dirk [17]	2020	6	Hormone-secreting pituitary adenoma		No
Nakassa et al. [19]	2017	7	Hormone-secreting pituitary adenoma	Out of the total hormone and non-hormone secreting cases, 18 patients received 25 mg of ICG and 22 received 12.5 mg.	No
Jeon et al. [15]	2018	4	Acromegaly; Cushing’s Disease; Null Cell with Hypogonadism	5 mg/kg 24 h before the operation	Yes
Cho et al. [12]	2018	14	Growth Hormone Secreting Adenoma; Adrenocorticotropic Secreting Adenoma; Prolactin Secreting Adenoma		No

**Table 2:** Demographic and Pathological Characteristics of ICG-Guided Pituitary Tumor Surgery Cohorts.

Characteristic	N	%
**Age**		
Mean/Median	50.5 years	-
Range	22–73 years	-
**Gender**		
Male	48	43.60%
Female	62	56.40%
**Pathology—FPT Types**		
Cushing’s Disease	19	17.30%
Acromegaly (GH-secreting)	24	21.80%
Prolactinoma	8	7.30%
Thyrotroph	1	0.90%
Gonadotropin-secreting	1	0.90%
**Pathology—Non-FPT Types**		
Non-functioning adenoma	24	21.80%
Craniopharyngioma	3	2.70%
Chordoma	4	3.60%
Rathke’s cleft cyst	3	2.70%
Meningioma	1	0.90%
Pituitary apoplexy	2	1.80%
Gangliocytoma	1	0.90%
**Total Cases**	110	-

## 4. Discussion

The use of ICG fluorescence in endoscopic pituitary adenoma surgery aims to improve surgical outcomes through enhanced tumor visualization. However, the literature on this topic, specifically regarding the utility of ICG in surgeries of FPT, remains limited and divided, with different approaches to ICG administration showing variable results. ICG is a water-soluble agent, inexpensive, approved by the Food and Drug Administration, and its safety profile is well established due to its historic use in measuring cardiac output and assessing hepatic blood flow and function. Adverse reactions to this agent are rare when given at standard doses below 0.5 mg/kg. When given intravenously, it has a plasma half-life of 3–5 min and is excreted in the bile within 15–20 min. ICG is cleared by the liver and remains unmetabolized in the bile. In the literature, complications following ICG administration are uncommon, and the very rare adverse events that have been documented include nausea and anaphylactic reactions, with an estimated incidence of fewer than 1 in 10,000 administrations. Patients with end-stage renal disease and those with isolated cases of coronary artery spasm may be at higher risk of developing an anaphylactic reaction with ICG [22]. Below, we have summarized the results from the literature we investigated to determine whether ICG fluorescence was found to significantly aid in the differentiation of FPTs from normal pituitary tissue.

### 4.1. Surgical Utilization of ICG in FPTs

Wang et al. conducted a study evaluating intraoperative ICG administration in a case of Cushing’s disease caused by a mixed pituitary adenoma and gangliocytoma [21]. The clinical effectiveness of this technique was demonstrated by the successful gross total resection of the tumor, which was confirmed by postoperative MRI. This led to the patient’s remission from Cushing’s disease, with their serum cortisol and ACTH levels dropping significantly on the second day post-surgery and a gradual improvement in all clinical symptoms. In another study conducted by Verstegen et al., evaluated low-dose ICG fluorescence in 10 patients, with a significant focus on functional tumors; this group included 6 FPTs (4 Cushing’s disease, 1 acromegaly, 1 prolactinoma) [20]. Their results demonstrated successful differentiation between the tumor and the normal gland in 9 out of 10 patients, including all the FPT cases. A significant finding was that the normal pituitary gland consistently showed a stronger fluorescent signal than the adenoma, with an average fluorescence contrast ratio of 1.5. This provided a reliable method for distinguishing tumor margins. The effectiveness of this technique was demonstrated by the fact that in two cases, one of which was an FPT, surgeons were able to perform the adenoma resection guided predominantly by NIR fluorescence, and repeated ICG injections helped confirm a complete resection. Additionally, the clinical effectiveness of the Verstegen et al. approach was demonstrated in the fact that all 6 patients with FPTs achieved biochemical remission postoperatively [20].

Subsequently, research done by Amano et al. and Muto et al. on a larger and diverse cohort highlighted that ICG administration effectively distinguished between adenoma and normal gland tissue in both FPTs and non-FPTs, although the quality of tumor visualization varied by tumor type [13,18]. Amano et al. evaluated 9 FPT (specifically somatotroph adenomas causing acromegaly) and 6 non-FPT cases [13]. Their results demonstrated successful differentiation between tumor and normal gland tissue in all 9 somatotroph adenoma cases. They pointed out a case of a 51-year-old woman with a GH-producing adenoma, where ICG use facilitated complete tumor resection, ultimately leading to remission of acromegaly, reflected by a postoperative GH level of only 0.14 ng/mL. Muto et al. studied ICG in 20 nonfunctioning adenomas, 3 somatotroph adenomas, and 2 lactotroph adenomas [18]. They observed 89% sensitivity for identifying tumors and 75% specificity for identifying the normal gland. A detailed subgroup analysis consisting of six non-FPTs, one somatotroph, and one lactotroph adenoma revealed that the Signal-to-Background Ratios (SBR) for these tumors ranged from 2.3 to 9.8, with higher ratios associated with better visualization. The two FPTs had ratios of 9.3 (somatotroph) and 2.3 (lactotroph). Notably, the SBR of 2.3 for the lactotroph adenoma was the lowest recorded among all 8 patients in the detailed subgroup analysis, suggesting that visualization of certain FPTs might pose greater challenges compared to non-FPTs due to their relatively lower fluorescence intensity, a finding that is also present in studies discussed further below. In their case report, the clinical effectiveness was demonstrated clearly, as the somatotroph adenoma patient achieved gross total resection and hormonal normalization, while the lactotroph adenoma patient had a subtotal resection due to cavernous sinus invasion, yet still experienced a dramatic drop in prolactin levels from 1402 ng/mL to 280 ng/mL at discharge [18].

Lee et al. confirmed the above-discussed subtypes of difference using a fusion imaging approach (five non-FPTs, one GH-secreting adenoma, and two adenomas with pituitary apoplexy) [16]. Their results also demonstrated stronger ICG uptake in non-FPT cases, as was clearly stated in Muto et al.’s work [18]. Nonetheless, the clinical effectiveness of this technique was evident as the same patient with the less visible GH-secreting adenoma underwent successful gross total resection, resulting in a significant postoperative reduction of basal GH to 5.7 ng/mL, compared to pre-operative levels of 51.23 ng/mL. Similarly, Inoue et al. demonstrated that integrating preoperative 3D modeling with intraoperative injection of 12.5 mg ICG fluorescence provides a comprehensive multimodal system that enhances surgical accuracy, even though they did not isolate ICG performance in FPTs specifically. But they concluded that combining preoperative 3D fusion models for planning with intraoperative ICG endoscopy for real-time guidance creates a useful system for improving surgical precision.

Both Lindner D and Nakassa et al. work highlighted the methodological variability and limitations of intraoperative ICG fluorescence for distinguishing pituitary adenomas from normal gland tissue [17,19]. Linder D evaluated 13 non-reactive pituitary adenomas, 6-hormone active, and one mucocele [17]. They showed that while ICG was highly effective for mapping vascular structures such as the internal carotid artery and cavernous sinus, it succeeded in differentiating tumor from normal tissue in only 15% of the cases, suggesting limited reliability for tissue-specific fluorescence. Similarly, Nakassa’a et al. analyzed 16 pituitary adenoma cases, out of which 8 were FPTs (7 unspecified hormone-secreting and 1 prolactin-secreting) [19]. Their results demonstrated that nearly all hormone-secreting adenomas (7 of 8) showed low fluorescence. The one exception was a single prolactin-secreting adenoma, which exhibited high fluorescence. Despite the generally low fluorescence in FPTs, the study concluded that a higher ICG dose was useful for differentiating tumors from adjacent structures [19]. The specific methodological factors within this study that may have contributed to these variable results will be analyzed in Section 6, as they confirm the need to revise existing ICG use protocols and standardize the more effective methodologies to achieve consistently better visualization and surgical outcomes.

Likewise, Jeon et al. and Cho et al. investigated the utilization of the second-window ICG (SWIG) technique for intraoperative visualization of pituitary adenomas and reported highly consistent findings related to its sensitivity [12,15]. Jeon et al. Studied 8 cases of pituitary adenoma, divided into 4 FPTs (two Cushing’s disease, one acromegaly, one causing hypogonadism) and 4 non-FPTs [15]. With this technique, they got 100% sensitivity and an average SBR of 3.9, showing no difference between FPT and non-FPT groups to identify potential residual tumors. Similarly, Cho et al. had 16 patients, with 9 FPTs (4 acromegaly, 2 Cushing’s, 2 prolactinoma, 1 thyrotroph) and 7 non-FPTs [12]. They concluded that ICG was a universally applicable agent for successfully visualizing all adenomas with an average SBR of 4.0 in the FPT group and 4.1 in the non-FPT group [12].

A preliminary analysis of remission rates across the reviewed studies demonstrates favorable clinical outcomes with both low-dose and high-dose ICG administration techniques. Low-dose, intraoperative protocols achieved notably high remission rates: Verstegen et al. reported 100% biochemical remission in all six FPT patients [20], Amano et al. documented complete hormonal normalization in a GH-producing adenoma with postoperative GH levels of 0.14 ng/mL [13], Muto et al. achieved hormonal normalization in a somatotroph adenoma and achieved a 80% reduction in prolactin levels (from 1402 ng/mL to 280 ng/mL) in a lactotroph adenoma despite subtotal resection [18], Lee et al. achieved an 89% reduction in basal GH levels (from 51.23 ng/mL to 5.7 ng/mL) [16], and Wang et al. achieved complete remission of Cushing’s disease with significant postoperative drops in cortisol and ACTH levels [21]. However, studies employing low dose intraoperative ICG under limited time-dependent interpretation protocols, such as Lindner D (15% differentiation success) and Nakassa et al. (low fluorescence in 7 of 8 FPT cases), demonstrate that technique standardization and adherence to precise timing protocols are critical for achieving consistent clinical outcomes [17,19]. In contrast, the high-dose preoperative SWIG technique achieved 100% sensitivity for tumor detection in both Cho et al. and Jeon et al. studies, with average signal-to-background ratios of 4.0 and 3.9, respectively, ensuring reliable tumor visualization across all FPT subtypes [12,15]. Although the SWIG studies we summarized did not report specific biochemical remission rates, the 100% sensitivity could enable surgeons to confidently identify all residual tumor tissue and achieve complete resection, which is the prerequisite for biochemical remission. The evidence we summarized, GH-secreting adenomas such as somatotrophs, appear particularly amenable to ICG guidance, with high tumor-to-background fluorescence ratios in the documented cases of gross total resection accompanied by postoperative hormonal normalization. In our findings, prolactinomas, as compared to lactotrophs, demonstrated lower fluorescence ratios, though some isolated reports of high signal in this tumor category exist. Importantly, the SWIG technique produces strong fluorescence across all FPT and non-FPT subtypes, whereas low-dose protocols using bolus ICG administration may favor adenomas such as somatotrophs.

**Figure 2:**
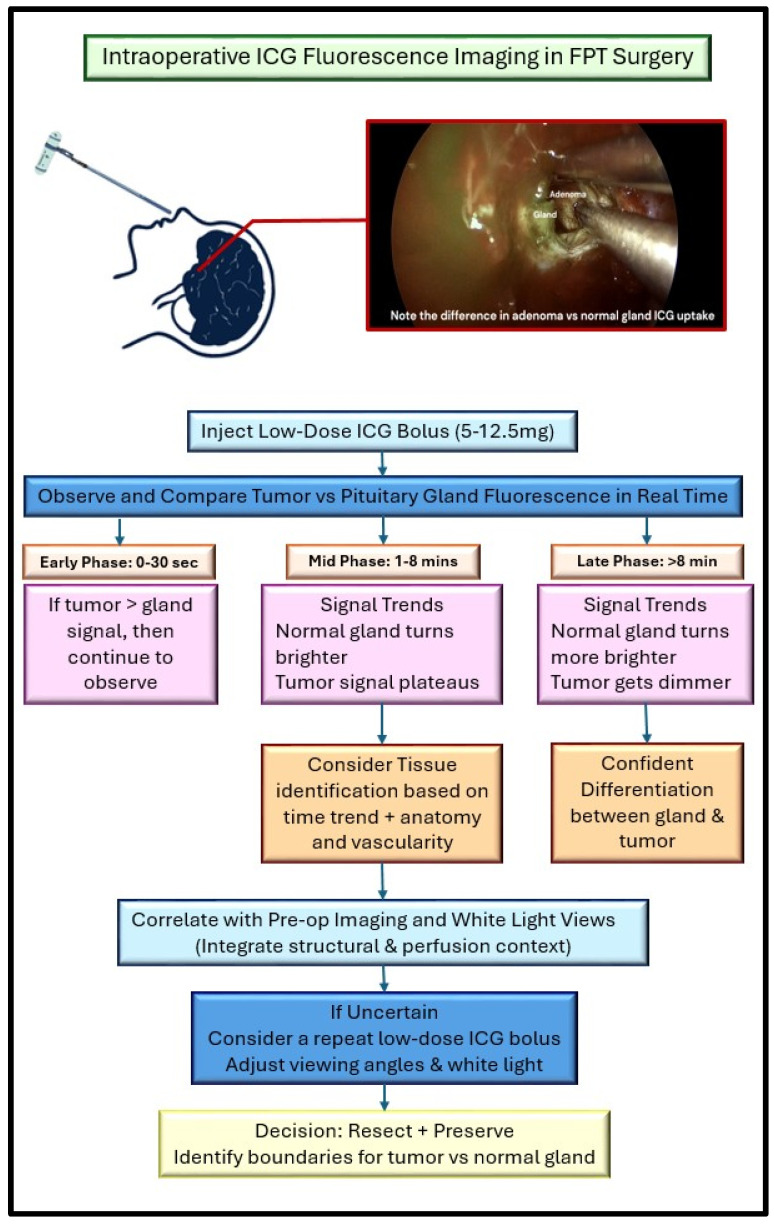
Stepwise interpretation algorithm for intraoperative bolus ICG fluorescence guidance during FPT surgery. This flowchart outlines a structured process to assist surgical teams in real-time interpretation of ICG signal dynamics, incorporating time-dependent perfusion patterns and tissue contrast behavior [20].

Intraoperative imaging techniques in pituitary surgery give neurosurgeons real-time information that helps make transsphenoidal procedures safer and more accurate (Figure 2). One of the most used tools is intraoperative MRI (iMRI), which provides detailed images of the tumor and nearby structures like the cavernous sinus, optic nerves, and cerebral fiber tracts, thereby improving intraoperative assessment of residual tumor and increasing the likelihood of gross-total resection [23]. However, iMRI requires a specifically designed operating room shielded from external magnetic fields and extends surgical duration by 10 to 30 min, during which the surgery must be paused, potentially reducing efficiency of the workflow and increasing the risk of acquiring surgery-related infections [24,25]. Despite iMRI’s excellent detailed anatomical imaging, it lacks real time functional feedback and cannot prevent neurological injury during the surgery. Intraoperative neuromonitoring addresses this limitation by providing continuous functional assessment that enables timely intervention to avoid irreversible neural damage, yet this one too does not enhance visualization of tumor vasculature. In contrast, ICG fluorescence offers immediate visualization of vascular neurosurgery and its role in expanding pituitary surgery [22]. To overcome the limitations of each modality, we propose actively integrating ICG fluorescence with intraoperative neurophysiological monitoring, combining dynamic perfusion assessment with continuous functional monitoring to provide complementary information not achievable by either modality alone.

### 4.2. ICG Dosing

The reviewed studies demonstrate that while ICG is a clinically effective tool for visualizing FPTs, its utility is not uniform. The variability in outcomes is largely attributable to the wide range of administration techniques employed. These methodologies can be broadly categorized into two distinct approaches: low-dose, intraoperative administration focused on real-time dynamics, and high-dose, preoperative administration (SWIG) focused on passive retention, which are discussed in the following sections.

#### 4.2.1. Low-Dose, Intraoperative Techniques: A Focus on Dynamic Differentiation

Most of the reviewed studies utilized a low-dose ICG injection during the surgical procedure. This approach relies on observing the dynamic differences in blood flow and vascular permeability between the highly vascular normal gland and the often less vascular adenoma. The specific methodologies and dosages varied significantly, which in turn influenced the results.

The most successful studies developed specific, time-dependent protocols to interpret these dynamics. Amano et al., using a very low dose of 6.25 mg, identified a crossover point around 8 min, where the initially brighter tumor becomes dimmer than the gland [14]. Similarly, Muto et al., using a 12.5 mg dose, defined a delayed window of 15–90 min where the normal gland was significantly brighter [19]. This time-dependent interpretation proved highly effective, leading to remission in all functional cases in the Amano et al. and Muto et al. studies [14,19]. Verstegen et al. also found success with repeated low-dose administrations of 5 mg (up to 15 mg total), noting that the normal gland consistently showed a stronger signal than the adenoma [21].

While the other studies highlighted the challenges of this approach, particularly the variable fluorescence of functional tumors. Wang et al. (12.5 mg) and Lee et al. (12.5 mg) both observed that their FPT cases showed relatively weaker ICG uptake compared to non-FPTs, even though differentiation was still successful [17,26]. Nakassa et al., who tested both 12.5 mg and 25 mg doses, directly addressed this by concluding that the higher 25 mg dose was better for differentiation, suggesting that a sufficient dose is critical, especially for tumors that have weak fluorescence [20]. Their observation of a median differentiation time of just 41 s also points to a very rapid, perfusion-based mechanism. Lastly, Inoue et al. (12.5 mg) proposed a hybrid solution, combining real-time ICG with preoperative 3D-CT/MRI fusion models to provide both anatomical and vascular guidance, compensating for any limitations of ICG alone [15].

The importance of a structured interpretation protocol is starkly illustrated in the research conducted by Lindner D [18]. Despite using intraoperative ICG, they reported a near-total failure to differentiate tumors from gland (with only 15% success). This result likely highlights a critical methodological point: without a specific time-dependent analysis protocol, the technique value collapses. The Lindner D study appears to have used ICG primarily as a traditional angiogram to map blood vessels while also expecting an immediate, obvious difference between the tumor and normal tissue [18]. By not reporting a timed observation strategy like Amano et al. or Muto et al., they likely missed the subtle, dynamic patterns essential for success [14,19]. This underscores that for low-dose ICG, the interpretation protocol is as crucial as the dye itself.

#### 4.2.2. High-Dose, Preoperative SWIG Technique: A Focus on Sensitivity

The SWIG technique, utilized by both Cho et al. and Jeon et al. in their respective studies, represents a completely different philosophy [13,16]. By administering a high dose of ICG (5 mg/kg) approximately 24 h preoperatively, it leverages the Enhanced Permeability and Retention (EPR) effect. This long waiting period allows the dye to passively leak from and become trapped within the tumor’s disorganized vasculature, while being cleared from healthy tissue. The result is a tumor that itself glows brightly, rather than relying on a comparison with the gland [13,16].

This method, as demonstrated across both studies, ensures that all tumors, including all FPTs, are reliably visualized with a strong fluorescent signal (average SBR of 3.9 and 4.0, respectively) [13,16]. The primary advantage of SWIG, confirmed by both research groups, is its 100% sensitivity. This means that if a tumor is present, it will glow. This, in turn, yields the technique’s most powerful clinical feature, which is a 100% Negative Predictive Value, a finding also consistent across both studies. For a surgeon, this provides an unparalleled all-clear signal, giving them absolute confidence that any non-glowing tissue is free of tumor and can be safely preserved. This is invaluable in FPT surgery, where preserving the healthy gland is critical for preventing iatrogenic hypopituitarism.

However, both studies also highlighted the technique’s major drawback: its high sensitivity comes at the cost of having very low specificity, which ranged from 20% (Jeon et al.) [13] to 25% (Cho et al.) [16]. They further suggested that expert matter knowledge of the surgeon can help in interpreting the glowing tissue at the margins. The same EPR effect that traps ICG in tumors can also occur in areas of inflammation or highly vascular normal structures such as the pituitary stalk, leading to a high rate of false positives. Therefore, as both studies conclude, a surgeon using SWIG cannot simply resect all glowing tissue; they must use their judgment to interpret these signals [13,16].

Clinical decision-making regarding the ICG administration technique can also be selected based on tumor subtype biology and size. Pituitary tumors vary significantly in vascularity and permeability, which may influence fluorescence behavior. ICG bolus administration provides real time assessment of vascular perfusion, whereas SWIG administered 24 h preoperatively relies on the enhanced permeability, retention effect and passive diffusion, allowing ICG microaggregates to accumulate within tumors that exhibit leaky or hypervascular architecture. The distinction is relevant in the case of pituitary adenoma subtypes with different vascular profiles. In a study conducted by Litvack et al., hormonally active adenomas, such as somatotroph which are densely granulated showed strong fluorescence, whereas null-cell adenomas exhibited minimal tumor fluorescence [22]. Observations suggesting that adenomas with higher vascularity or invasive nature may benefit from the SWIG administration of ICG which can enhance the tumor gland contrast and identify regions of invasion. The EPR effect might not be needed with SWIG in case of smaller and less vascular pituitary adenomas, making intraoperative bolus administration of ICG sufficient [13,16].

## 5. Conclusions

Ultimately, the choice of ICG administration technique represents a fundamental trade-off, the importance of which is magnified in the context of FPTs. For these tumors, achieving a true, complete resection is essential, as even microscopic amounts of residual tissue can continue to secrete excess hormones, leading to persistent disease and the need for adjuvant therapies.

With this imperative in mind, the two approaches offer distinct solutions. The low-dose, intraoperative methods provide a highly specific, real-time guide for dissection, ideal for surgeons skilled in interpreting dynamic patterns to meticulously separate the adenoma from the gland. The high-dose, preoperative SWIG technique, while used for guidance throughout the procedure, offers its most powerful advantage during the final margin assessment. Its 100% sensitivity ensures all potential tumor tissue glows, while its perfect 100% Negative Predictive Value provides a definitive “all-clear” signal for non-glowing areas. This allows a surgeon to confidently preserve healthy tissue and conclude the operation with great confidence that no tumor is left at the dark margins.

The collective evidence suggests that while no single method is perfect, a thoughtful application of either technique, tailored to the specific surgical goal, be it precise real-time guidance or final margin confirmation, can significantly enhance the surgeon’s ability to achieve the complete resection necessary for curing FPT surgery.

## 6. Limitations

While this review highlights the growing utility of ICG fluorescence in FPT surgeries, several limitations must be acknowledged due to the early stage of research in this field. The foremost issue is the small and heterogeneous sample sizes across studies, which range from single case reports to limited cohorts of up to 40 patients, with relatively fewer FPT cases. This restricts statistical validity and prevents meaningful meta-analysis, particularly given the biological and vascular differences among hormone-secreting subtypes that may influence ICG fluorescence. Equipment availability also poses a challenge as specialized NIR-capable systems are costly and often limited to a few cases per day, which may introduce selection bias and hinder wider clinical adoption. Additionally, the cost of ICG administration itself represents a significant financial consideration, as it adds to the overall surgical expense; while enhanced visualization may reduce the need for revision surgeries and adjuvant therapies, the cost-effectiveness of this approach requires further economic analysis across diverse healthcare settings.

Moreover, methodological variability, arising from differences in imaging systems, ICG dosing, and reporting metrics, limits comparability between studies. Some report on qualitative observations, while others employ fluorescence contrast or signal-to-background ratios, impeding standardized outcome assessment. From a safety standpoint, while ICG is generally well-tolerated, allergic reactions have been documented in patients with iodine sensitivity or prior adverse reactions to contrast agents [27]. These reactions, though rare, represent an important clinical consideration that necessitates careful preoperative screening protocols and the availability of emergency medications during administration. The lack of standardized safety monitoring and reporting across studies further complicates the assessment of ICG’s true adverse event profile in this population.

For future research, concrete priorities such as a prospective head-to-head trial comparing the low-dose dynamic of ICG versus SWIG protocols should be tested and developed to establish relative efficacy. Moreover, efforts should focus on defining standardized dosing and imaging parameters, such as timing of administration and intraoperative visualization criteria, to enable reproducible protocols across centers. It should also include comprehensive cost-effectiveness analyses and systematic safety surveillance to establish the true clinical and economic value of ICG fluorescence in FPT surgery.

## Data Availability

No new data were created or analyzed in this study.
